# Acylation – A New Means to Control Traffic Through the Golgi

**DOI:** 10.3389/fcell.2019.00109

**Published:** 2019-06-12

**Authors:** Andreas M. Ernst, Derek Toomre, Jonathan S. Bogan

**Affiliations:** ^1^Department of Cell Biology, Yale School of Medicine, Yale University, New Haven, CT, United States; ^2^Section of Endocrinology and Metabolism, Department of Internal Medicine, Yale School of Medicine, Yale University, New Haven, CT, United States

**Keywords:** Golgi, palmitoylation, acylation, anterograde transport, Golgi bypass, membrane traffic

## Abstract

The Golgi is well known to act as center for modification and sorting of proteins for secretion and delivery to other organelles. A key sorting step occurs at the *trans*-Golgi network and is mediated by protein adapters. However, recent data indicate that sorting also occurs much earlier, at the *cis*-Golgi, and uses lipid acylation as a novel means to regulate anterograde flux. Here, we examine an emerging role of S-palmitoylation/acylation as a mechanism to regulate anterograde routing. We discuss the critical Golgi-localized DHHC S-palmitoyltransferase enzymes that orchestrate this lipid modification, as well as their diverse protein clients (e.g., MAP6, SNAP25, CSP, LAT, β-adrenergic receptors, GABA receptors, and GLUT4 glucose transporters). Critically, for integral membrane proteins, S-acylation can act as new a “self-sorting” signal to concentrate these cargoes in rims of Golgi cisternae, and to promote their rapid traffic through the Golgi or, potentially, to bypass the Golgi. We discuss this mechanism and examine its potential relevance to human physiology and disease, including diabetes and neurodegenerative diseases.

## Introduction

A major function of the Golgi is to receive, sort and modify proteins and lipids and to deliver these cargoes to new locations [e.g., the plasma membrane (PM), endo-lysosome, or back to endoplasmic reticulum (ER)]. Impaired coordination of this highly orchestrated assembly line can result in abnormal glycosylation (Climer et al., 2015; Fisher and Ungar, 2016) or a virtual “traffic jam.” In neurons, Golgi dysfunction is associated with numerous diseases such as Parkinson’s, Alzheimer’s, and Huntington’s diseases, as well as with cognitive disorders (Glick and Nakano, 2009; Bexiga and Simpson, 2013; Rabouille and Haase, 2015). Altered Golgi function in other cell types may also contribute to disease. Yet, how the mammalian Golgi sorts proteins and lipids remains poorly understood.

## Organization of the Mammalian Golgi

In mammalian cells the Golgi looks like a perinuclear crescent or “ribbon” by light microscopy and as a “stack” of pancake-like cisternae by three-dimensional electron microscopy (Ladinsky et al., 1999; Huang and Wang, 2017; Martinez-Martinez et al., 2017), with the *cis* side closest to the ER. The cisternae are stacked by GRASP proteins (Zhang and Wang, 2015) and individual Golgi “mini”-stacks are laterally linked to form an extensive Golgi ribbon (Wei and Seemann, 2017; Makhoul et al., 2018). The crescent-shaped ribbon is often several micrometers long and highly convoluted. It contains, on average, five cisternae with a thickness of 50 nm each, which are separated by intercisternal spaces of 5–15 nm (Ladinsky et al., 1999). This organization of the Golgi differs drastically from that found in lower eukaryotes: yeast Golgi is neither stacked nor linked and within the cisternae, the Golgi enzymes exchange dynamically to cause maturation of the enclosed cargo (Losev et al., 2006; Glick and Nakano, 2009). These differences in Golgi structure underscore one of the biggest and most debated riddles in cell biology: How does the Golgi function to sort anterograde traffic?

Whereas for retrograde traffic there is a clear consensus that COPI vesicles mediate retrograde flux, for anterograde traffic multiple models have been proposed, including those of vesicular transport, cisternal maturation, and others (Patterson et al., 2008; Jackson, 2009; Pfeffer, 2010; Glick and Luini, 2011). Central to this longstanding debate is the question of whether cisternae within the mammalian Golgi ribbon mature (i.e., dynamically exchange their enzymes), or whether the enzyme composition of a given cisterna is stable, with cargo passing through static successive layers via vesicular/tubular carriers (Glick and Luini, 2011). Two longstanding challenges in addressing this question are that (1) current imaging cannot visualize live Golgi dynamics at sufficient resolution to unambiguously distinguish between these models (which may require live cell imaging at tens of nanometers in 3D), and (2) the machinery used for anterograde traffic is debated (e.g., Do COPI vesicles carry only retrograde traffic or do they act in both directions?). Arguably, the only current consensus is that there is no consensus. But might there also be other means to drive cargo forward?

## A *Cis*-Face Problem: How to Distill Anterograde Cargo From Retrograde Proteins and Lipids?

A remarkable requirement of Golgi function upstream of intra-Golgi transport lies in the segregation of anterograde from retrograde cargo and lipids (Glick and Nakano, 2009). Indeed, one of the largest remaining questions in Golgi biology is exactly how sorting of anterograde from retrograde cargo (ER resident proteins, lipids) is achieved upon Golgi entry. While glycosylation is a highly coordinated and critical function of the Golgi, there is little evidence that it is a driving force for sorting (Gomez-Navarro and Miller, 2016). But how much sorting is there? This has been addressed by comparing the total amount of anterograde membrane that leaves the ER via COPII vesicles to the amount of new membrane actually required to sustain cell growth (Barlowe and Helenius, 2016; Bottanelli et al., 2017). Notably, about 90% of the membrane has to be recycled to the ER and, in light of this large retrograde backflow, the incoming cargo has to be extensively concentrated to achieve an efficient rate of anterograde net movement.

The question remains as to whether anterograde sorting of cargo at the Golgi is an active process (i.e., mediated by unknown signals), or whether a net anterograde flux is achieved simply by the selective retrograde retrieval of lipids and proteins upon entry into the Golgi. More simply, is active sorting required to move cargo forward, or not? More is understood about the retrograde traffic machinery, which has been extensively characterized and appears to consist mainly of COPI vesicles and tubular Rab6- connections. Both act in conjunction with the selective retrieval of retrograde cargo and depend on basic amino acids that serve as concentration and retrieval signals (White et al., 1999; Duden, 2003; Barlowe and Helenius, 2016). How sorting of anterograde cargo is achieved at the *cis* face of the Golgi is much less clear. One obvious concept is that there may be an anterograde amino acid sorting signal. Here, it was recently proposed that acidic residues on the cytoplasmic tail of a model transmembrane cargo, vesicular stomatitis virus glycoprotein (VSV-G) could promote anterograde routing at the Golgi (Fossati et al., 2014). Nevertheless, the lack of such residues in the cytoplasmic tails of multiple viral spike proteins (HA, NA, and GP), as well as mammalian integral PM resident proteins, suggests that such signals do not act generally in anterograde sorting, but rather are specific to VSV-G at COPII exit sites in the ER (Votsmeier and Gallwitz, 2001).

### The *Cis*-Golgi Is a Hot Spot for Protein Palmitoylation

Hints that some type of lipid-based anterograde sorting signal might exist appeared in the 1980s, when it was shown that efficient intra-Golgi cargo transport requires a specific type of activated lipid, palmitoyl-CoA (Glick and Rothman, 1987). Furthermore, it was discovered that the hydrolysis and transfer of palmitoyl-CoA is necessary for both vesicle budding and fission, and that its presence is needed in donor Golgi membranes (Pfanner et al., 1989; Ostermann et al., 1993). These data positioned the acylation reaction at the *cis*-Golgi. However, despite uncovering a potential role of palmitoyl-CoA in anterograde Golgi sorting, the underlying molecular mechanism could not be elucidated.

## Palmitoylation – Lipidation of Proteins as a Molecular Switch?

In recent years, palmitoyl-CoA has increasingly been recognized for its importance in the fields of developmental and cell biology, in microbiology, and in neuroscience (El-Husseini Ael et al., 2002; Linder and Deschenes, 2007; Fukata and Fukata, 2010; Demers et al., 2014). In addition to its role in the synthesis of membrane lipids (Blom et al., 2011), palmitate is attached covalently and post-translationally to several hundreds of proteins (Blanc et al., 2015). These protein lipidation (or fatty acylation) reactions can occur on three types of different amino acids: (1) on cysteine residues (forming thioester linkages, S-palmitoylation), (2) on serine/threonine residues (forming oxyester linkages, O-palmitoylation), or (3) on primary amino groups (forming amide linkages, N-palmitoylation) (Jiang et al., 2018). Among these distinct types of acylation, S-palmitoylation stands out, as the thioester-bond is easily reversible and can thus act as a two-way toggle. Further, acylation has an inherent biophysical propensity to dynamically alter the properties of the modified protein, including the oligomerization state within a membrane or the targeting of soluble proteins to membranes (Smotrys and Linder, 2004). As such, S-acylation is well-positioned to function as a molecular and biophysical switch. For clarity, while the fatty acid S-acylation is typically palmitate, other fatty acids such as stearate (C18) and unsaturated variants can be used.

## DHHC S-Palmitoyltransferases Are Found in Key Positions Across the Secretory Pathway

In mammals, the enzymes responsible for protein S-acylation are a family of 23 proteins, termed protein-acyl transferases or PATs. These integral membrane proteins typically contain four to six transmembrane domains and a cysteine-rich domain (CRD) within a cytoplasmic loop. A conserved Asp-His-His-Cys (DHHC) tetrapeptide motif is located within the CRD and is the active site of each “DHHC” enzyme (Fukata and Fukata, 2010; Greaves and Chamberlain, 2011; De and Sadhukhan, 2018; Rana et al., 2019). The fact that the active site is positioned at the cytoplasmic face dictates that unlike N-palmitoylation, which takes place in the lumen of the Golgi (Buglino and Resh, 2008; Jiang et al., 2018), S-acylation occurs exclusively in the cytoplasm. Acylation of a substrate protein is thought to be mediated by a two-stage mechanism: (1) auto-acylation of a single or multiple Cys residue(s) within the CRD of the DHHC enzyme by Acyl-CoA to form an enzyme-acyl thioester moiety, and (2) the Cys residue from a substrate protein attacks the enzyme-acyl intermediate to form a substrate-acyl product (Rana et al., 2019). Notably, integral membrane proteins frequently have Cys residues immediately adjacent to their transmembrane anchors (Aicart-Ramos et al., 2011), and recent crystallographic data position the active site of the PAT near the cytoplasmic leaflet (Rana et al., 2018).

Unlike most other posttranslational modifications, S-acylation does not seem to require a consensus motif. Rather, it appears that the juxtamembrane positioning of the substrate Cys residue close to the PAT active site is required for acylation of the substrate (Rodenburg et al., 2017). How such a spatial positioning is achieved to promote the acylation of soluble proteins is even less clear.

A separate set of enzymes, called acyl-protein thioesterases (APTs), mediates the de-acylation of S-palmitoylated proteins (Vartak et al., 2014; Lin and Conibear, 2015; Won et al., 2018). Interestingly, APTs are soluble proteins that are recruited to Golgi membranes, in part by S-palmitoylation, and that are further able to undergo auto-depalmitoylation. Recent work shows that one isoform, APT1, resides on mitochondria and that the S-palmitoylation of mitochondrial proteins is dynamically regulated (Kathayat et al., 2018). Thus in distinct cellular membranes, acylated cargo proteins are positioned in vicinity to enzymes that remove this modification, so that the abundance of acylated proteins may be maintained under homeostatic control (Vartak et al., 2014).

Given that there are 23 DHHC PAT isoforms in mammals, several important questions naturally arise. Where are these acylation/de-acylation machineries localized? Do PATs exhibit differential expression patterns across different tissues? Are they present in different stages of development or stages of cell proliferation? Ohno et al. set out to address these points and examined both the subcellular location of overexpressed PAT isoforms as well as their expression levels in different tissues (Ohno et al., 2006). Strikingly, the majority of DHHC isoforms appeared to localize exclusively or partially to the Golgi, while others exhibited a distinct localization to the ER, the PM, or to vesicular-tubular structures. Furthermore, a tissue-specific expression pattern was observed for multiple isoforms, e.g., DHHCs 11, 19, and 20 appear exclusively in the testis, while many were ubiquitously expressed and only appear to be absent in distinct tissues.

### S-Palmitoylation Is a Committed Step for Anterograde Transport at the *Cis*-Golgi

A recurring theme in the literature is the correlation between S-palmitoylation and the affinity of membrane proteins for sphingolipid/cholesterol-containing membranes (Levental et al., 2010; Salaun et al., 2010; Ernst R. et al., 2018). The observations that DHHC isoforms localize to distinct positions within the secretory pathway and are present in distinct tissues at specific levels suggest that S-palmitoylation serves a purpose beyond that of targeting proteins to cholesterol-rich microdomains. However, the strong concentration of DHHC isoforms in the Golgi could be interpreted as a requirement of protein lipidation to achieve compatibility with the complex (and cholesterol-sphingolipid rich) membranes encountered at the *trans*-Golgi network and beyond. A gradient of sphingolipids and cholesterol exists within the Golgi, with the *trans*-Golgi/TGN exhibiting the highest concentrations of both lipid classes (Van Meer et al., 2008). If S-palmitoylation serve the purpose of sorting anterograde cargo to “raft-like” membranes, this is presumably where DHHC enzymes should be localized.

## Concentration of DHHC Isoforms in the *Cis*-Golgi

Recently, the intra-Golgi localization of DHHC PATs was investigated in a quantitative manner using exogenously expressed tagged constructs (Ernst A.M. et al., 2018). 17 out of 23 DHHC PATs exhibited significant overlap with endogenous Golgi markers, and 9 localized to the Golgi exclusively: DHHCs 3, 7, 9, 11, 13, 15, 17, 21, and 22. Strikingly, 6 out of 9 DHHCs exhibited strong co-localization with endogenous *cis*-Golgi markers (DHHCs 3, 7, 13, 17, 21, and 22), while the remaining 3 were positioned at the *trans*-Golgi (DHHCs 9, 11, and 15). Interestingly, all *cis*-Golgi-localized DHHCs are expressed in most tissues, while those detected at the *trans*-Golgi are highly tissue-specific (Ohno et al., 2006). If sorting into “raft-type” microdomains might be a main function of protein S-palmitoylation, then the observations prompt the question of why the entire ubiquitously expressed pool of Golgi DHHC PATs is found in the *cis* [a subcompartment with very low cholesterol and sphingolipid contents (Jackson et al., 2016)]. Notably, cell-free system studies of minimal components needed for anterograde Golgi transport demonstrated that palmitoyl-CoA in *cis* Golgi donor membranes greatly facilitated anterograde cargo transport and budding (Glick and Rothman, 1987; Pfanner et al., 1989; Ostermann et al., 1993). Together, this suggests that palmitoylation may do more than only sorting proteins to “raft” membrane domains.

## S-Palmitoylation Induces Anterograde Sorting of Membrane Cargo

**FIGURE 1:**
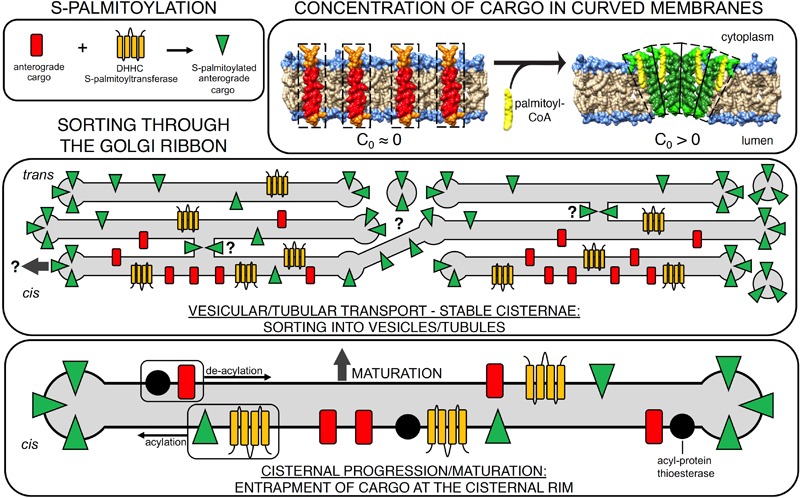
S-palmitoylation acts as a switch to sort anterograde cargo through the Golgi. Membrane cargo entering the Golgi undergoes rapid S-palmitoylation by *cis*-Golgi resident DHHC protein-acyl transferases (top left). Acylation of the membrane protein results in a change in the spontaneous curvature of cargo (C_0_ > 0), generating curvature stress (top right). This can also concentrate the acylated cargoes. Middle panel: effect of S-acylation on cargo transport in the vesicular/tubular transport model (stable cisternae). Acylation causes the cargo to be stabilized in regions of higher curvature, e.g., in (1) tubular intercisternal connections (Trucco et al., 2004), (2) the highly curved and fenestrated cisternal rim (Orci et al., 1997), and (3) in tubular or vesicular carriers budding thereof (Glick and Rothman, 1987; Pfanner et al., 1989; Ostermann et al., 1993), putatively providing a mechanism for Golgi bypass (arrow). Lower panel: effect of S-acylation on cargo transport in the cisternal progression/maturation model: S-palmitoylation triggers an entrapment of cargo at the cisternal rim. This facilitates the extraction of retrograde lipids and cargo and reduces the inclusion of anterograde cargo in the retrograde carriers. Thioesterase activity allows for cycles of lateral diffusion back to the cisternal center and re-acylation by DHHCs.

Independently, Ernst A.M. et al. (2018) employed a clickable analog of palmitate, alkyne-palmitate, to identify in mammalian cells the major site of S-acylation activity. Pulse-chase based metabolic labeling revealed a rapid and specific incorporation of palmitate into the *cis*-Golgi. This incorporation depended on activation of the probe with CoA and resulted in thioester linkages to proteins within Golgi membranes other than the aforementioned DHHC PATs; specificity was validated by Triacsin C (a competitive inhibitor of Acyl CoA synthetase) and hydroxylamine-sensitivity of the palmitate labeling *in situ* and of proteins on SDS-PAGE (neutral hydroxylamine cleaves thioester linkages present of S-acylated proteins, but not oxyester linkages formed from incorporation of palmitate into lipids). In pulse-chase experiments, the S-palmitoylated proteins partitioned over time from the *cis*- to the *trans*-Golgi, in a strictly anterograde fashion, with no signal appearing in the ER. The cargo then appeared at the PM, but this did not occur if DHHC enzyme activity was impaired. To identify the *cis*-Golgi PAT responsible for the apparent anterograde cargo routing, candidate Golgi-localized PATs (DHHCs 3, 7, 9, 11, 13, 15, 17, 21, 22) were overexpressed and probed for catalysis of anterograde routing of S-palmitoylated proteins. Only the closely related DHHCs 3 and 7 were capable of catalyzing the rate and extent of anterograde transport of bulk S-acylated proteins. Concordantly, the model S-acylated substrates VSV-G and transferrin receptor were probed for a differential partitioning through the Golgi as a function of their acylation status (importantly, both cargoes are classical “non-raft” markers, ruling out a sphingolipid/cholesterol-dependency of the sorting). Strikingly, while the rate of *entry* into the Golgi was identical for wildtype and mutant cargoes, transport *through* the Golgi was slowed when these cargo proteins were not acylated, strongly suggesting that S-acylation represents a sorting event, routing them efficiently along an anterograde track. These data suggest that the biochemical requirement for palmitoyl-CoA for *in vitro* reconstitution trafficking assay detected in reports 30 years prior (Glick and Rothman, 1987; Pfanner et al., 1989) stemmed from S-palmitoylation of the model cargo VSV-G employed in the cell-free system, and that no other unknown cofactors were involved in modulating partitioning of VSV-G form donor to acceptor Golgi membranes. In search for an explanation of how S-palmitoylation modulated the anterograde routing of cargo, alkyne-palmitate-based metabolic labeling was combined with electron tomography. In agreement with an earlier observation that indicated VSV-G preferentially accumulated at the cisternal rims (Orci et al., 1997), the authors found that bulk S-palmitoylated proteins are indeed strongly enriched in the highly curved perimeters of the cisternal rim, which consists of tubules and fenestrated sheet-like elements (Ladinsky et al., 1999). In order to test whether sorting to areas of high curvature results directly from S-acylation of the cargo, model acylated transmembrane peptides were probed for a partitioning between flat and curved membranes *in vitro*. Strikingly, acylation of the peptides resulted in a strong partitioning into highly curved membranes, to an extent in line with the increase in anterograde transport observed for trafficking of model acylated cargoes through the Golgi. Together, the data strongly support a model whereby S-acylation directly modulates the biophysical property of the cargo, resulting in its increased partitioning to the cisternal rim of *cis*-Golgi membranes, which in turns facilitates its anterograde routing (see Figure 1).

## Additional Examples for Anterograde Routing of S-Acylated Membrane Cargo

### Linker for Activation of T Cells (LAT)

Hundt et al. (2009) investigated the trafficking of LAT, a dually S-palmitoylated protein that is a crucial signaling molecule for T-cell receptor-based stimulation of T-cells (Hundt et al., 2009). Anergic T cells lack palmitoylation of LAT, resulting in a reduction in Tyr phosphorylation and activation of PLCγ1 (Ladygina et al., 2011). Hundt et al. demonstrated that when LAT is not S-palmitoylated, its coupling to sphingolipid/cholesterol-rich membranes is not affected, but rather results in LAT’s accumulation in the Golgi, with negligible levels detected at the PM. Further, LAT is in the family of transmembrane adaptor proteins (TRAPs), and the additional family members LIME and NTAL/LAB also were shown to require S-palmitoylation for efficient export to the PM (Stepanek et al., 2014). Thus, anterograde routing of TRAPs via S-palmitoylation at the Golgi emerges as a requirement for T-cell function.

### β-Adrenergic Receptors (AR)

β-adrenergic receptor (AR) isoforms 1–3 are all S-palmitoylated proteins, but these isoforms exhibit different sites of acylation. Recently, Adachi and colleagues investigated mammalian β_3_AR, and observed that a distinct site (Cys-153) is crucial for proper targeting of the receptor to the PM, while other sites (Cys-361/363) impact its stability at the PM (Adachi et al., 2019). These observations prompt the hypothesis that within the AR family, different sites of S-palmitoylation are employed to toggle between states, and may control AR targeting to different cellular locations, modulating its surface expression and hence downstream signaling.

### GABA Type a Receptors (GABA_A_Rs)

GABA receptors are well-established S-acylated PM residents. Based on studies using overexpressed DHHCs, they were postulated to be palmitoylated by DHHCs 3 and 7 (Fang et al., 2006). Subsequently, Kilpatrick et al. (2016) identified DHHC3 as a specific PAT of the γ2 subunit of GABA_A_Rs through knockout of either DHHC3 or 7 in mice (Kilpatrick et al., 2016). Whereas knockout of DHHC7 had no effect on GABA_A_R trafficking in neurons, DHHC3 KO neurons exhibited drastically reduced levels of GABA_A_R γ2 at synapses, impacting synaptic function. Most remarkably, and in line with the identification of DHHCs 3 and 7 as ubiquitous regulators of protein sorting at the *cis*-Golgi (Ernst A.M. et al., 2018), knockout of the individual PATs resulted in only marginal effects, while a double knockout of DHHCs 3 and 7 resulted in perinatal lethality of mouse embryos, emphasizing the importance of these PATs and sorting at the *cis*-face of the Golgi for general cell function.

### Glucose Transporter 4 (GLUT4)

Insulin stimulates glucose uptake in fat and muscle cells by causing the exocytic translocation of GLUT4 to the PM. Although this had previously been considered exclusively as a post-Golgi process, more recent data make clear that recycled GLUT4 accumulates in a pool of small (∼50 nm diameter) vesicles that reside near the ERGIC and *cis*-Golgi compartments, in association with TUG, Golgin-160, and ACBD3 (Acyl-CoA Binding domain-containing protein 3, also known as GCP60) (Xu et al., 2011; Bogan, 2012; Orme and Bogan, 2012; Belman et al., 2015). Upon insulin stimulation, these vesicles are mobilized by TUG cleavage, and they are proposed to traffic to the cell surface by an unconventional secretion pathway that bypasses the Golgi stack (Xu et al., 2011; Bogan, 2012; Habtemichael et al., 2018). GLUT4 is S-acylated and the PAT responsible for this modification was recently identified (Du et al., 2017). Both DHHCs 3 and 7 bound to GLUT4, but only silencing of DHHC7 abolished GLUT4 acylation, leading to the conclusion that DHHC7 acts as a specific PAT for GLUT4. Other proteins that cotraffic with GLUT4 or that regulate this process are also palmitoylated in adipocytes (Ren et al., 2013a). Importantly, the ability of insulin to stimulate GLUT4 translocation was impaired by knockdown or knockout of DHHC7 and by mutation of the palmitoylated Cys residue in GLUT4 (Ren et al., 2015; Du et al., 2017). Thus, it may be that S-acylation is required to concentrate the GLUT4 in the highly curved membranes of the insulin-responsive vesicles.

## S-Palmitoylation Confers Membrane Proteins a “Sorting-Competent” State

How might S-palmitoylation of proteins induce partitioning to the highly curved rims of Golgi cisternae? As noted above, S-palmitoylation occurs exclusively at the cytoplasmic leaflet of endomembranes, frequently on one or two adjacent sites and at juxtamembrane positions of integral membrane proteins. It is known that the c*is*-Golgi cisternae are stacked by the action of GRASP65 proteins, which leads to a significant flattening of membranes in the stacked area (Ladinsky et al., 1999). Upon S-palmitoylation, the acylated proteins would exhibit a local mass excess in the cytoplasmic leaflet of the membrane, concomitant with an asymmetric hydrophobic Z-profile (i.e., an increase in spontaneous curvature/transition from cylindrical to conical profile); see top right panel of Figure 1. In support of this concept, early studies on erythrocytes demonstrated that drugs intercalating into the cytoplasmic leaflet induced a morphological change from a flat and disc-like to spherical morphology (Sheetz and Singer, 1974). In the Golgi, the tightly stacking proteins in the central disk region would inhibit such a morphological transition – therefore, the local mass excess in the cytoplasmic leaflet due to S-palmitoylation is expected to induce curvature stress, forcing acyl chains in the cytoplasmic leaflet to potentially form locally curved clusters. This stress could presumably induce the observed partitioning toward the positively curved cisternal rims, where S-palmitoylation-induced curvature stress would be released (Figure 1). This reasoning suggests that S-palmitoylation serves as a biophysical switch and sorting signal at the *cis* Golgi, to extract cargo proteins from planar membranes and concentrate them at the cisternal rims.

The cisternal rim is fenestrated and comprises a network of tubular-vesicular elements, referred to as the “non-compact zone/region” (Ladinsky et al., 1999). A significant fraction of PM resident proteins (>15%) are predicted to be S-acylated (Ernst A.M. et al., 2018). Yet, the data nonetheless raise the question how anterograde cargo that lack this sorting signal can be efficiently sorted to the cisternal rim. The diffusive flux along the gradient generated by S-palmitoylated proteins would have the potential to drag along non-acylated proteins. In line with this hypothesis, overexpression of *cis*-Golgi DHHCs resulted in an increased flux of (soluble) secretory cargo, and even impacted non-acylated cargo to a lower but significant extent (Ernst A.M. et al., 2018). Multiple additional scenarios can also be envisioned, and could contribute to spontaneous curvature-based sorting of proteins at the Golgi: (1) conicity of the hydrophobic moiety (the membrane anchor) acquired through hetero-oligomerization or protein folding, as observed for the polytopic membrane channel KvAP, which exhibits affinity for areas of high curvature without being lipidated (Aimon et al., 2014), (2) heterooligomerization of palmitoylated and non-palmitoylated proteins, and (3) coagulation of integral S-palmitoylated proteins and luminal proteins via lectins, e.g., the mannose-specific lectin VIP36 that is present at the *cis* Golgi (Fullekrug et al., 1999).

A further consideration is that S-acylation-induced sorting of membrane proteins may apply to intracellular organelles other than the Golgi. At the ER, notably, S-palmitoylation of the ER-resident Calnexin alters its localization toward mitochondria-associated membranes (MAM), which exhibit a high extent of positive curvature (Lynes et al., 2012). Supportively, access of the ER-thioredoxin TMX to MAM also requires S-acylation (Lynes et al., 2012). However, S-acylation of Calnexin was also suggested to regulate access to sheet-like structures (Lakkaraju et al., 2012), which might not be a contradiction given that a recent study detected nanoholes within ER-sheets (that would give rise to curvature, Schroeder et al., 2019). Additionally, the endosome-localized DHHC15 was suggested to concentrate cargo for efficient uptake by retromer (Mccormick et al., 2008). Thus, S-palmitoylation may be a ubiquitous mechanism to cause the partitioning of integral membrane proteins into regions of high curvature.

From an evolutionary standpoint, S-palmitoylation represents a widespread post-translational modification that is conserved from yeast to mammals, plants, and even encountered in multiple parasites (Lam et al., 2006; Roth et al., 2006; Brent et al., 2009; Hemsley et al., 2013; Hodson et al., 2015). The fact that virtually all spike proteins of enveloped viruses are S-palmitoylated, regardless of whether or not their viral exit sites are enriched in sphingolipids/cholesterol (e.g., Influenza A NA, VSV-G, Ebola GP), further emphasizes the evolutionary pressure toward maintaining this putative anterograde sorting signal to maximize the efficiency of their anterograde routing at the Golgi. Possibly, palmitoylation may also induce curvature at the PM, which may be relevant for budding of membrane, in addition to targeting soluble and membrane proteins (Li et al., 2017; Rana et al., 2019; Wang et al., 2019).

### Controlled Access of Soluble Proteins to the *Cis*-Golgi S-Palmitoylation Machinery

Two members within the DHHC PAT family, the huntingtin-interacting proteins DHHC 13 and 17, particularly stand out because of their architecture. They contain long amino-terminal ankyrin-repeats, which were shown to bind to specific soluble substrate bearing corresponding ankyrin-binding motifs (Lemonidis et al., 2015). Among their substrates are the critical neuronal proteins MAP6 and CSP, but also the ubiquitous t-SNAREs in the SNAP25 family (Greaves et al., 2010; Lemonidis et al., 2014). Interestingly, Lemonidis et al. (2014) demonstrated that binding of these substrates to DHHCs 13 and 17 does not (13) or only marginally (17) induces their acylation, whereas DHHCs 3 and 7 are not able to bind the proteins but efficiently mediate their acylation. The fact that DHHCs 3, 7, 13, and 17 all localize to the *cis*-Golgi strongly suggest cooperativity among the PATs in this subcompartment, with DHHCs 13 and 17 serving to recruit these proteins to the *cis*-Golgi, where they are subsequently S-palmitoylated by DHHCs 3 and/or 7 in a concerted, two-stage process.

As noted above, several proteins involved in the regulation of GLUT4 translocation are palmitoylated, and some of these may also be recruited as soluble proteins onto highly curved membranes (Ren et al., 2013a,b; Du et al., 2017). In unstimulated cells, the insulin-responsive vesicles are trapped in association with ACBD3, which binds palmitoyl-CoA (Belman et al., 2015; Yue et al., 2019). The related protein ACBD6 facilitates N-myristoylation of substrate proteins by cooperating with N-myristoyltransferase enzymes (Soupene and Kuypers, 2019). Whether ACBD3 may participate in palmitoylation of soluble proteins is not known, although in general ACBD family members have diverse roles in lipid-modification and metabolism pathways (Chen et al., 2012; Nazarko et al., 2014; Soupene et al., 2016) and in Golgi structure (Yue et al., 2017; Liao et al., 2019).

Data also suggest that the accumulation of a pool of small, insulin-responsive vesicles may be impaired during the development of type 2 diabetes, which may result from excess membrane diacylglycerols and sphingolipids (Garvey et al., 1998; Maianu et al., 2001; Czech, 2017; Habtemichael et al., 2018; Petersen and Shulman, 2018). We speculate that these excess lipids might potentially disrupt the palmitoylation-based sorting mechanisms discussed above and thus contribute to attenuated insulin action.

### Outlook

#### Enrichment of S-Palmitoylated Cargo in Vesicular-Tubular Carriers Versus Entrapment in Maturing Cisternae at the Golgi

S-palmitoylation-induced sorting of cargo to the cisternal rim fits well with the long-proposed role of COPI vesicles as anterograde carriers (Rothman, 2014) in addition to their well-established role as retrograde carriers (Brandizzi and Barlowe, 2013). Artificially introducing stable adhesions (“staples”) between Golgi cisternae does not impair anterograde transport, showing that anterograde transport primarily occurs from the highly curved rims (Lavieu et al., 2013), where COPI vesicles bud (Orci et al., 1986). When mitochondria are engineered to invade the Golgi by affording them adhesion to its cisternae, these organelles dissect the cisternae apart and immobilize them (Lee et al., 2014). Nonetheless, anterograde transport continues (Dunlop et al., 2017), implying a small diffusible carrier. Independently, COPI vesicles, visualized by super-resolution microscopy, carry VSV-G protein between separated Golgi areas in fused cells and account for most of the cargo transported by this route (Pellett et al., 2013). We propose that palmitoylation-driven anterograde sorting bears the potential to help resolve the long-standing conundrum of how one coat could mediate two fates (Pelham, 1994). Retrograde cargo is selected into COPI vesicles by classic receptor-dependent binding (such as KDEL – KDEL receptor or KKXX – coatomer (White et al., 1999; Duden, 2003; Barlowe and Helenius, 2016)). We can envision a physical-chemical, membrane-intrinsic process by which S-palmitoylated anterograde cargo spontaneously clusters within curved regions to the exclusion of other proteins, including retrograde cargo and their receptors. The same COPI coat could then pinch off distinct anterograde and retrograde vesicles (Orci et al., 1997) from these differentiated regions of membrane.

An alternative interpretation, along the lines of a cisternal maturation model for intra-Golgi transport (Casler et al., 2019; Kurokawa et al., 2019), is that S-palmitoylation-induced sorting of cargo to the rim serves the purposes of redistributing the cargo while the cisternae mature (i.e., enzyme contents change). Potentially through the action of APT-mediated acylation-deacylation cycles, the de-palmitoylated cargo would lose their stable anchoring at the cisternal rim and partition back to the (flat) center of the cisterna. There, the cargo would engage in additional rounds of acylation by DHHCs, and through its partitioning back and forth across the cisterna, its chances of being processed by glycosyltransferases might increase. However, a challenge here is to explain how palmitoylated cargo would traffic through the maturing Golgi faster than non-palmitoylated cargo, as observed (Ernst A.M. et al., 2018). One would need to invoke that only the non-palmitoylated cargo partially traffic retrograde, for which there is currently scant evidence.

A third option would be a “fast-track” through the Golgi mediated by intercisternal continuities. VSV-G translocation through the Golgi was reported to induce intercisternal connections (Trucco et al., 2004), and additional types of GTPase-dependent tubules were detected on the Golgi (Park et al., 2015; Bottanelli et al., 2017). S-palmitoylation could here putatively provide a mechanism to allow partitioning into these highly curved connections between cisternae (Figure 1). To address this will require visualization of sufficiently large areas of the Golgi, with specificity, at very high resolution. This may be possible with new advances in super-resolution imaging (Huang et al., 2016) and with large volume electron microscopy approaches such as FIB-SEM (Kizilyaprak et al., 2019).

## Future Steps: Visualizing DHHC Platforms

Acylation at the *cis*-face of the Golgi emerges as an important mechanism for routing of several proteins to the PM. Given that multiple DHHC PATs are capable of cooperating to achieve S-palmitoylation of specific substrates (Lemonidis et al., 2014; Abrami et al., 2017), coupled with the fact that several of these isoforms are present only in distinct tissues (Ohno et al., 2006), indicates that screening DHHC libraries to identify “specific” PATs for a protein of interest is not adequate. PATs are emerging as finely adjusted enzyme assemblies rather than isolated entities, and thus their spatial context (location within the same cisterna and across cisternae) has to be studied – ideally at endogenous levels and by means of super-resolved light and electron microscopy. These nano-assemblies are expected to vary in composition across different tissues and during different developmental stages/states of cell proliferation. We hypothesize that DHHC platforms have the potential to provide a mechanism for fine-tuning the flux of specific substrates from the Golgi to the PM, thereby controlling the availability and timing of receptors, channels, and other types of cargo at the cell surface.

With S-acylation as a marker for anterograde cargo in hand, live-cell and super-resolution microscopy-compatible orthogonal labeling strategies need to be developed to better visualize trafficking of anterograde cargo as a class (as opposed to individual proteins with varying properties) – on the nanoscale and on timescales that allow to observe complete transport/maturation cycles across the mammalian Golgi. These advances would allow investigations to reopen the exploration of putative differences in the mechanism of intra-Golgi transport between lower and higher eukaryotes, and furthermore would enable detailed studies of acylation-controlled protein export from the Golgi in response to external stimuli. Such studies could elucidate important fundamental principles, as well as insights into mammalian physiology and disease.

## Author Contributions

AE, DT, and JB researched the relevant literature, and conceived and wrote the manuscript.

## Conflict of Interest Statement

The authors declare that the research was conducted in the absence of any commercial or financial relationships that could be construed as a potential conflict of interest.
